# Mitochondrial Quality Control in Bovine Oocyte Maturation: Mechanisms, Challenges, and Prospects for Enhancing Reproductive Efficiency

**DOI:** 10.3390/ani15132000

**Published:** 2025-07-07

**Authors:** Yi-Ran Zhang, De-Jun Xu

**Affiliations:** Chongqing Key Laboratory of Herbivore Science, College of Animal Science and Technology, Southwest University, Chongqing 400715, China; rio12345@email.swu.edu.cn

**Keywords:** mitochondrial quality control, oocyte, maturation, epigenetics

## Abstract

Oocyte maturation represents a fundamental biological process in bovine reproduction, establishing the physiological basis for fertilization and early embryonic development while critically determining the propagation of improved varieties and breeding efficiency. The roles of MQC in reproduction have gained substantial scientific attention. The proper maturation of oocytes fundamentally depends on adequate mitochondrial functionality. However, the intrinsic regulatory mechanisms governing MQC during bovine oocyte maturation remain incompletely characterized. Here, we discuss the most recent progress on the molecular mechanisms and roles of mitochondrial fission/fusion, biogenesis, and mitophagy in MQC. Building upon the mechanistic foundations of MQC in bovine oocyte maturation, this review identifies key mitochondrial-targeted supplements with potential applications in enhancing oocyte quality. Furthermore, we evaluate epigenetic influences on mitochondrial regulatory networks through mitochondrial–nuclear communication. Finally, we discuss the challenges in elucidating mitochondrial quality control mechanisms during oocyte maturation and propose corresponding strategies to address these obstacles. Integrating mechanistic insights, this review proposes strategies to enhance in vitro culture systems and identify oocyte quality markers, providing valuable insights for optimizing in vitro production (IVP) of bovine embryos and enhancing reproductive efficiency.

## Simple Summary

The past decades have witnessed significant advancements in live-cell suborganelle imaging technologies and multi-omics approaches, providing powerful analytical tools to investigate mitochondrial functional dynamics during bovine oocyte maturation. Nevertheless, persistent challenges in mitochondrial dysfunction and compromised oocyte maturation quality continue to constrain mammalian reproductive efficiency and biological research progress. Emerging evidence suggests that dysregulation of mitochondrial quality control (MQC) networks may underlie these abnormalities by disrupting critical physiological processes, including energy metabolism, meiotic regulation, and intracellular signaling pathways. This review elucidates the mechanistic regulation of bovine oocyte maturation through MQC pathways, offering novel perspectives for improving reproductive efficiency by targeting mitochondrial function.

## 1. Introduction

Oocyte quality stands as a pivotal determinant of female reproductive success in cattle, critically governing fertilization, embryonic development, and postnatal growth trajectories [1,2]. During maturation, oocytes undergo sequential biological milestones, including cytoplasmic maturation, chromosomal recombination, and meiotic progression, to yield fully functional gametes. Mitochondria serve as essential organelles in oocytes through multiple mechanisms. Primarily, they sustain energy demands during oocyte maturation via continuous ATP production through integrated metabolic pathways, particularly oxidative phosphorylation coupled with the tricarboxylic acid cycle [3]. Furthermore, mitochondria physically and functionally interact with the endoplasmic reticulum to modulate intracellular calcium fluxes. These calcium ions act as critical signaling mediators that coordinate meiotic resumption and cell cycle progression [3]. Concurrently, mitochondrial ATP generation inherently produces reactive oxygen species (ROS), where excessive accumulation may induce oxidative damage through protein denaturation and lipid peroxidation [4]. Mitochondria also regulate cellular fate through metabolic intermediates that influence signaling cascades, exemplified by cytochrome C release initiating apoptotic pathways [4]. Notably, tricarboxylic acid cycle metabolites directly mediate epigenetic modifications: acetyl-CoA facilitates histone acetylation while α-ketoglutarate modulates DNA demethylation through enzymatic regulation.

Mitochondrial quality control (MQC) constitutes a central regulatory axis governing oocyte maturation, coordinating ATP biogenesis, selective elimination of damaged organelles, preservation of mtDNA integrity, and dynamic mitochondrial reorganization. Earlier studies focused on nutritional influences or individual MQC components. However, recent research demonstrates that mitochondrial biogenesis, dynamics (particularly the fusion/fission balance), and selective autophagy (mitophagy) work synergistically during bovine oocyte maturation. Notably, follicular development synchronizes morphological and quantitative adaptations in oocytes and granulosa cells (GCs), with mitochondrial abundance, spatial distribution, and functional integrity directly dictating developmental outcomes [5,6]. Dysregulated mitochondrial dynamics and compromised mitophagy not only impair ovarian architecture but also exacerbate oxidative stress in GCs, while emerging evidence links these perturbations to diminished oocyte quality and broader reproductive health consequences [7,8]. This synthesis elucidates the molecular interplay of MQC mechanisms governing bovine oocyte maturation, proposing targeted strategies to enhance in vitro maturation systems, mitigate age-related fertility decline, and optimize breeding protocols for improved livestock reproductive efficiency and herd sustainability. This review systematically discussed the impact of MQC mechanisms on bovine oocyte maturation through molecular regulatory networks, synthesizes key regulatory pathways identified in current research, and proposes potential strategies to enhance mitochondrial health for oocytes.

## 2. Oocyte Maturation

Oocytes maturation progresses through three critical stages: germinal vesicle breakdown (GVBD), metaphase I (MI), and metaphase II (MII). The initial phase involves the growth and proliferation of primordial oocytes, during which they gradually acquire the morphological and structural features of mature gametes. This process encompasses two parallel mechanisms: nuclear maturation (involving chromosomal segregation) and cytoplasmic maturation (marked by organelle reorganization and energy accumulation). Studies have identified multiple signaling pathways, including the mTOR [9,10] and AMPK pathways [11,12], as well as translational control mechanisms, that regulate oocyte development. Embryos derived from oocytes with inhibited mTOR signaling during ovulation exhibit insufficient energy supply and reduced developmental potential [13]. Notably, in vitro maturation studies of bovine oocytes demonstrate that mTOR inhibition enhances energy metabolism, highlighting its critical role in balancing oocyte bioenergetics. Current evidence confirms the essential role of mTOR in sustaining mitochondrial oxidative capacity. mTOR inhibition rapidly disrupts carbon utilization and mitochondrial metabolism in leukemia cell lines. Leukemic cells treated with rapamycin exhibit impaired mitochondrial function, resulting in preferential reliance on aerobic glycolysis over mitochondrial respiration for energy production [14]. Investigations demonstrate that mTOR exerts metabolic control through a Bcl-xl-VDAC1 complex localized at the mitochondrial outer membrane. In skeletal muscle systems, rapamycin suppresses the expression of mitochondrial transcriptional regulators (PGC-1α, ERRα, NRF), consequently reducing mitochondrial gene expression and oxygen consumption. Pharmacological mTOR inhibitors induce mitochondrial hyperfusion, aberrant branching, and disrupted fission processes via mTORC1 axis decoupling, ultimately triggering apoptotic cell death [14,15,16]. Specifically, bovine oocytes treated with Juglone and matured in vitro showed downregulation of the PI3K/AKT/mTOR pathway and its downstream signals [17]. This was manifested by autophagy induction with significantly increased levels of LC3B and beclin-1; increased DNA damage with overexpression of DNA damage-specific markers (8-Oxoguanine; 8-OxoG); significant downregulation of glycolysis-specific genes (PFK1 and GLUT1); and significantly reduced expression of ATP synthesis genes (ATPase8, ATP5F1B) in oocytes [18]. Normally, the translation initiation factor eIF4E (cap-binding protein) undergoes progressive phosphorylation during meiotic maturation of bovine oocytes. At the GVBD-mid I transition, eIF4E phosphorylation coincides with a significant increase in protein synthesis, whereas at mid II, oocyte protein synthesis declines to levels comparable to GV oocytes despite full eIF4E phosphorylation [19]. eIF4E phosphorylation timing strongly correlates with MAP kinase activation. Furthermore, the eIF4E-specific repressor 4E-BP1 forms a complex with eIF4E; enhanced eIF4E/4E-BP1 complex formation in mid-II oocytes coincides with reduced translation rates at this stage, suggesting 4E-BP1 involvement in inhibiting cap-dependent translation in mid-II bovine oocytes [20]. Interestingly, the study found that 4E-BP1 becomes progressively phosphorylated from the GVBD stage to the MII stage, while the mTOR inhibitor Torin2 irreversibly arrests bovine oocyte maturation at the MI stage. Torin2 treatment inhibits translation by enhancing 4E-BP1 binding to eIF4E [21], thereby suppressing 4E-BP1 phosphorylation at Thr37/46 in a reversible manner. However, no significant differences were observed in mTOR expression levels or phosphorylation between IVM and Torin2-treated groups. Collectively, the roles of mTOR and eIF4E in bovine oocyte maturation require further investigation [22].

Chromosomal recombination and meiosis are central to nuclear maturation, wherein crossovers’ formation and synaptonemal complex assembly ensure accurate chromosomal segregation through physical chiasma stabilization. Mitochondria cooperate with the meiotic spindle to achieve asymmetric division. The presence of formin2 (FMN2) around oocytes induces the aggregation of short actin bundles derived from endoplasmic reticulum vesicles at the spindle periphery, which subsequently drives mitochondria associated with the spindle, promoting their redistribution and facilitating spindle migration [23,24]. Recent evidence links mitochondrial activity fluctuations to meiotic competence: developmentally competent oocytes exhibit two distinct peaks of mitochondrial activation (prior to meiotic resumption and before maturation completion), while suboptimal oocytes show only one activation event preceding maturation [25,26]. Overexpression of mitochondrial fusion protein MFN2 increases mitochondrial density near the spindle in mouse oocytes without significantly altering mitochondrial membrane potential (MMP) or ROS production [27]. This suggests that MFN2 influences nuclear maturation of oocyte through the mitochondrial pathway. Paradoxically, excessive mitochondrial accumulation around the spindle inhibits spindle movement and chromosomal separation, causing metaphase I arrest [27]. Mitochondrial Rho-GTPase 1 (MIRO1) regulates mitochondrial transport and mitophagy during meiosis. MIRO1 deficiency disrupts meiotic resumption and polar body extrusion in mouse and pig oocytes. Mechanistically, MIRO1 interacts with microtubule-organizing center (MTOC)-associated motor proteins to regulate Aurora Kinase A (Aurora A) and kinesin KIF11, thereby promoting spindle assembly [28]. Furthermore, MIRO1 coordinates with dynamin-related protein 1 (DRP1), Parkin, and lysosome-associated membrane glycoprotein 2 (LAMP2) to regulate mitochondrial dynamics and quality control during oocyte maturation [28,29].

The cytoplasmic maturation of oocytes is a prerequisite for normal fertilization, with mitochondrial redistribution and cortical granule maturation serving as key indicators. Mitochondria primarily generate ATP via oxidative phosphorylation (OXPHOS) through an electron transport chain (ETC) comprising five complexes (I–V) on the inner mitochondrial membrane [30]. The proton gradients established by respiratory chain complexes I, III, and IV power ATP synthase-catalyzed phosphorylation, whereas complexes I and III serve as primary sites for superoxide generation through electron leakage at flavin mononucleotide (FMN) clusters and Q-cycle intermediates, respectively [31]. This ROS overproduction triggers mitochondrial membrane potential collapse and mtDNA mutations via oxidative chain reactions [32,33]. Mitochondrial activation and spatial reorganization directly influence cytoplasmic maturation, with mitochondrial integrity determining oocyte developmental competence and fertilization success. In bovine oocytes, active mitochondria transition from cortical localization at the GVBD stage to cytoplasmic dispersion by MII. This redistribution is absent in developmentally incompetent oocytes, where mitochondria remain peripherally restricted [34,35].

**Figure 1:**
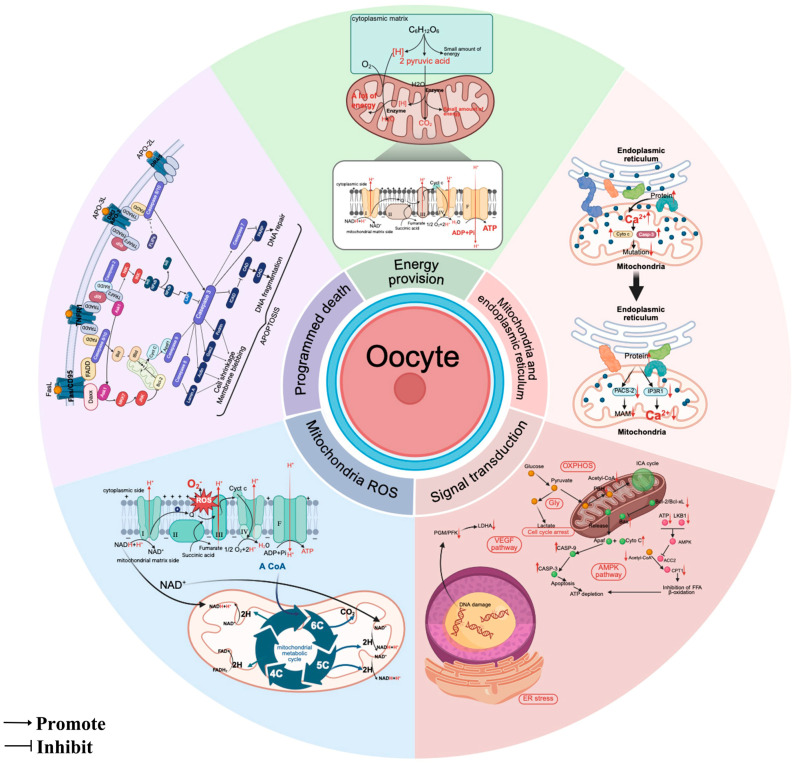
Mitochondrial essential functions in oocyte maturation: encompassing bioenergetic supply, coordinated calcium regulation, ROS dynamics, cellular signaling, and apoptotic control. Up arrow: Upregulating; Down arrow: Downregulating. Created in BioRender. https://www.biorender.com/ (accessed on 10 April 2025).

Emerging evidence demonstrates that epigenetic remodeling accompanies oocyte maturation. Histone H4 acetylation and dynamic expression of histone deacetylases (HDACs) are observed during bovine oocyte maturation [36]. Mitochondrial metabolites act as enzymatic cofactors or inhibitors. For instance, DNA methyltransferases transfer methyl groups from S-adenosylmethionine (SAM) to cytosine residues, forming 5-methylcytosine (5mC), which can be passively diluted or actively removed by ten-eleven translocation (TET) enzymes in an α-ketoglutarate (α-KG)-dependent manner [37]. Metabolic interventions reducing the α-KG/succinate ratio increase global H3K27 and DNA methylation in mouse embryonic stem cells, while α-KG supplementation rescues embryos with defective epigenetic reprogramming [38]. These findings collectively demonstrate that oocyte maturation integrates cellular growth, nuclear–cytoplasmic coordination, and mitochondrial metabolic adaptation, with profound implications for improving oocyte quality in reproductive biotechnology (Figure 1).

Most experiments did not specify oxygen conditions, indicating they were conducted under ambient atmospheric oxygen. Additionally, studies revealed an interaction between oxygen tension and oocyte density that increases ROS production under specific combinations, subsequently affecting bovine oocyte in vitro maturation quality [39]. This may be attributed to the elevated oxidative stress induced by reactive oxygen species (ROS) under hyperoxic conditions, which in turn triggers abnormalities in mitochondrial autophagy regulation, ultimately impairing the maturation quality of oocytes. Future studies should further elucidate the mechanism underlying the relationship between distinct oxygen concentrations and bovine oocyte maturation.

## 3. Mitochondrial Quality Control

MQC is an integrated regulatory network responsible for monitoring mitochondrial quality, which is crucial for maintaining mitochondrial homeostasis and functionality in cells. MQC orchestrates mitochondrial homeostasis through coordinated processes, including mitochondrial biogenesis, fission and fusion, proteolysis, autophagic degradation, and selective clearance of damaged mitochondrial inner membranes. Within the mitochondrial quality control system, mitochondrial biogenesis represents a critical compensatory mechanism activated in oocytes experiencing elevated energy demands or cellular stress. This process involves continuous mtDNA replication, restoration of mtDNA integrity and electron transport chain components, coupled with the replacement of dysfunctional mitochondria with functional organelles to maintain adequate ATP production. The regulation of mitochondrial biogenesis demonstrates intricate coordination between mtDNA and nuclear genomic mechanisms [40,41]. Mitochondrial dynamics encompasses the perpetual cycle of fission and fusion events that dynamically regulate organellar morphology, size, and intracellular distribution to adapt to fluctuating energy requirements and cellular signaling demands [42,43]. Mitochondrial proteostasis, mediated through specialized proteolytic systems, constitutes an essential defense mechanism against oxidative damage, protein misfolding, and electron transport chain deficiencies. Within the mitochondrial matrix, a collaborative network of proteases, including the CLPXP complex and Lon protease homolog (LONP), orchestrates the selective degradation of aberrant proteins while regulating metabolic enzyme turnover [44]. The quality control cascade culminates in mitophagy, a selective autophagic process that ensures cellular homeostasis through targeted elimination of irreparably damaged mitochondria, thereby preserving optimal mitochondrial function and genomic integrity [45,46].

Under oxidative stress, studies demonstrate that SOD2 acetylation significantly increases mtROS production. Honokiol supplementation markedly elevates SIRT3 protein levels and restores the NaF-reduced SOD2 acetylation form in Western blotting (WB) analysis (*p* < 0.05) [47]. SIRT3 inhibition reduces deacetylated superoxide dismutase 2 (SOD2) levels, triggering mitochondrial ROS-induced oxidative stress-mediated apoptosis. This disrupts redox balance during oocyte maturation and causes mitochondrial damage [47,48]. Isorhamnetin activates the PI3K/Akt pathway, counteracting oxidative stress and mitochondrial dysfunction significantly (*p* < 0.05) to promote maturation [49].

Mitochondrial metabolites also critically influence oocyte maturation. In bovine oocytes, pyruvate conversion to acetyl-CoA is modulated by sodium dichloroacetate (DCA), which elevates H3K9ac levels, alters transcriptional activity, and potentially compromises oocyte quality [50]. Pyruvate metabolic disturbances during maturation activate β-oxidation, disrupt mitochondrial metabolism, and reduce mRNA content [50,51]. L-carnitine facilitates fatty acid β-oxidation by transporting activated fatty acids into mitochondria via carnitine palmitoyltransferase I (CPT1), generating acetyl-CoA for ATP production [52]. Supplementation with L-carnitine in IVM media improves embryo outcomes by providing essential cofactors for fatty acid utilization. Recent findings highlight its role in balancing acetyl-CoA/CoA ratios, sustaining glucose metabolism, enhancing mitochondrial energy output, and alleviating endoplasmic reticulum stress by shuttling or eliminating toxic palmitate [53]. Additionally, L-carnitine improves mitochondrial distribution uniformity in mature oocytes by scavenging ROS [54,55].

**Figure 2:**
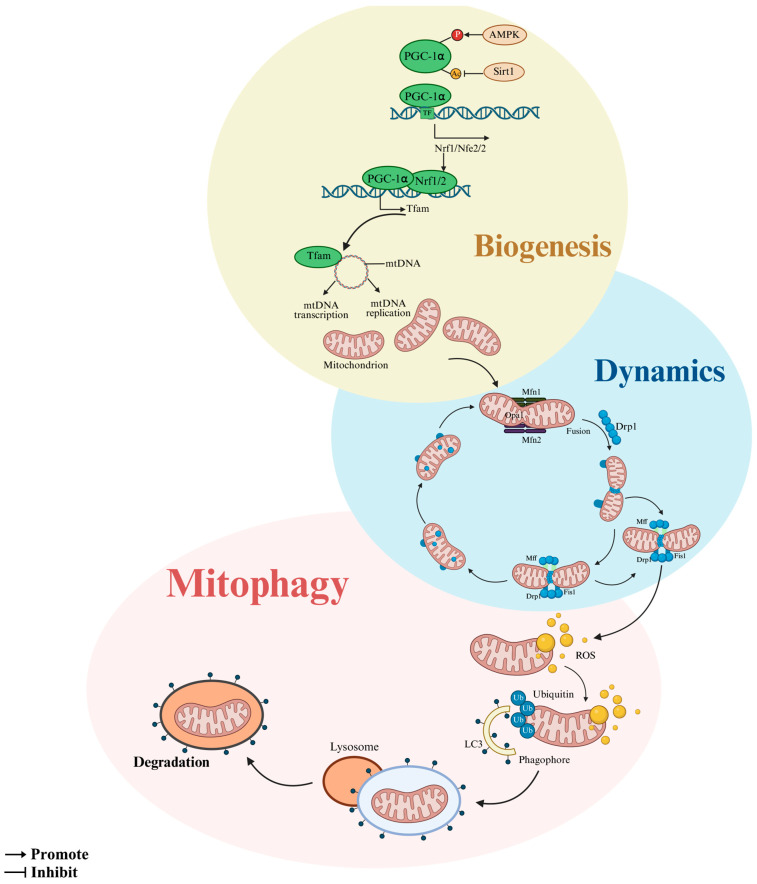
Mitochondrial quality control pathways in oocytes. Mitochondrial biogenesis, mitochondrial dynamics, and mitophagy are closely interrelated. Created in BioRender. https://www.biorender.com/ (accessed on 10 April 2025).

Collectively, the MQC network operates dynamically to maintain mitochondrial integrity. Supplementation with resveratrol or melatonin enhances mitochondrial activity, upregulates biogenesis and fission/fusion significantly (*p* < 0.05), and mitigates dysfunction in damaged mitochondria. While nutritional studies elucidate MQC mechanisms via pathways such as PGC1α/NRFs [56,57], PINK1/Parkin [58,59], cAMP-PKA [60], PI3K/AKT [61,62], and Drp1 [63], most evidence derives from murine and porcine models. The intrinsic molecular mechanisms governing MQC in bovine oocytes remain poorly characterized, warranting further investigation (Figure 2).

## 4. Mitochondrial Biogenesis and Its Molecular Role in Oocyte Maturation

Mitochondrial biogenesis serves as a homeostatic mechanism to sustain mitochondrial quantity by continuously replenishing damaged mitochondria with fully functional counterparts [64]. As a core determinant of mitochondrial function, this process plays a pivotal role in oocyte cytoplasmic maturation. Mitochondrial biogenesis involves a complex cascade of events, requiring coordinated activation of multiple factors under diverse biological stimuli. During meiotic resumption and cytoplasmic maturation, oocytes demand substantial ATP production, primarily supplied through mitochondrial oxidative phosphorylation. Throughout meiotic maturation, mitochondria dynamically redistribute within the oocyte, accumulating around the spindle apparatus during the first meiotic division [65]. This spatial coordination between mitochondrial distribution and meiotic spindle integrity is critical, as ATP deficiency disrupts spindle morphology and meiotic progression. Experimental evidence confirms that silencing mitochondrial-associated genes severely compromises spindle architecture and meiotic completion [65,66].

**Figure 3:**
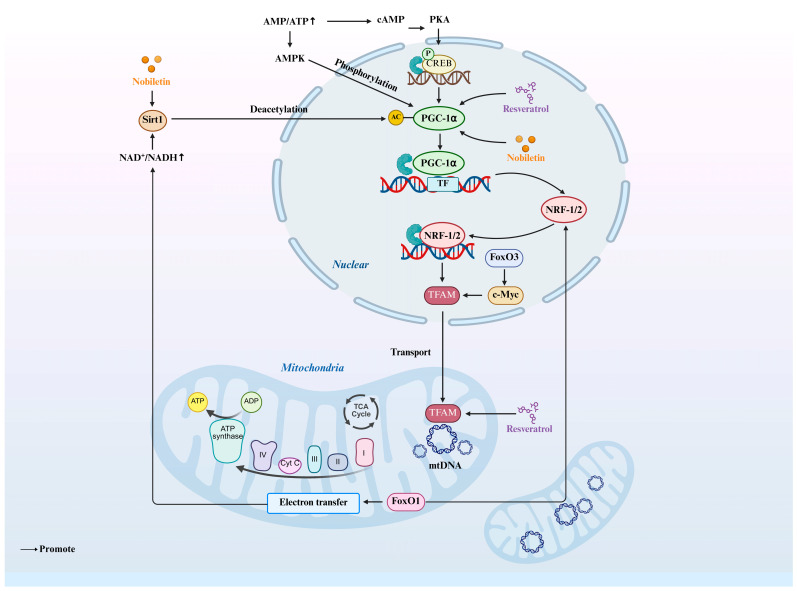
The major signaling pathways for mitochondrial biogenesis. PGC-1α and NRF1/2 serve as core transcriptional regulators that orchestrate mitochondrial DNA replication and electron transport chain assembly through transcriptional control of TFAM. Up arrow: Upregulating Created in BioRender. https://www.biorender.com/ (accessed on 10 April 2025).

Current research focuses on key signaling pathways, notably peroxisome proliferator-activated receptor gamma coactivator 1-alpha (PGC-1α), a master transcriptional coactivator that drives mitochondrial biogenesis through upregulating nuclear respiratory factors (NRF1 and NRF2) and mitochondrial transcription factor A (TFAM) (Figure 3) [67,68]. This pathway enhances mitochondrial DNA replication, electron transport chain (ETC) component synthesis, and ATP generation. Mature oocytes exhibit elevated mtDNA content, underscoring the necessity for stringent regulation of mitochondrial replication and biogenesis during maturation. Notably, resveratrol depletion significantly upregulates critical biogenesis-related genes such as PGC-1α and TFAM [69,70]. In porcine oocytes, SIRT1 activation has been shown to enhance mitochondrial degradation and biogenesis, thereby improving oocyte quality. The study revealed that TFAM, PGC1α, and PINK1 expression levels were all higher in the treatment group compared to the untreated group [71,72]. Recent studies reveal that rotenone suppresses SIRT1 levels during in vitro maturation (IVM) of porcine oocytes, exacerbating mitochondrial dysfunction, increasing mitophagy, and impairing mitochondrial biogenesis, ultimately disrupting oocyte maturation [73]. Early research proposed that mitochondrial transcription factor A (TFAM) exhibited higher expression in morphologically superior oocytes regardless of follicular size, suggesting its association with developmental competence [26,74]. Subsequent investigations demonstrate that SIRT2 inhibition downregulated TFAM, perturbing mitochondrial biogenesis and dynamics during oocyte maturation [75,76]. Pre-IVM treatment with C-type natriuretic peptide (CNP) upregulated mitochondrial biogenesis-related genes, promoted mitochondrial biogenesis, and enhanced cytoplasmic maturation of oocytes [67,68]. In porcine oocytes, 10 μM of nobiletin enhanced mitochondrial biogenesis by elevating SIRT1 and PGC-1α protein levels. This intervention led to increased active mitochondrial populations, mtDNA copy number, mitochondrial membrane potential, and ATP output, thereby improving mitochondrial functionality and promoting in vitro maturation. While multiple studies in bovine oocytes and other species correlate elevated mtDNA content with enhanced developmental competence, the molecular basis underlying this association remains ambiguous [51,77,78]. This persistent knowledge gap necessitates further investigation to reconcile discrepant findings in the existing literature.

Additionally, FOXO1 and FOXO3a serve as critical signaling components in mitochondrial biogenesis. Research indicates that AMPK collaborates with SIRT1 to modulate the expression of energy metabolism-related genes in skeletal muscle. AMPK elevates cellular nicotinamide adenine dinucleotide (NAD^+^) levels, thereby enhancing SIRT1 activity and promoting deacetylation and functional regulation of downstream targets such as FOXO1 and FOXO3a [79]. In neuronal contexts, SIRT2 regulates mitochondrial biogenesis through deacetylation-mediated activation of transcription factors FOXO1, FOXO3a, and TFEB, along with the coactivator PGC-1α [80]. Overall, FoxO1 and FoxO3a function as suppressors of mitochondrial biogenesis through distinct molecular mechanisms. In renal tubular epithelial cells, FoxO1 exerts its inhibitory effect by blocking the formation of the CREB-CBP-P300 transcriptional complex. This disruption subsequently reduces PGC-1α expression and impairs mitochondrial biogenesis. In contrast to FoxO1, FoxO3-mediated transcriptional suppression functions through a distinct pathway independent of PGC-1 family members or NRF1. This inhibitory mechanism specifically involves targeting c-Myc, a key transcription factor that activates nuclear-encoded mitochondrial genes via direct binding to the TFAM promoter [81].

In summary, although the PGC1α/NRF1/2 axis, TFAM-mediated regulation, and FOXO1/FOXO3A signaling are well-established biomarkers for mitochondrial biogenesis, with recent advances in understanding mTOR [82,83] and p53 [84] signaling during bovine oocyte maturation, the molecular crosstalk between mitochondrial biogenesis and MQC networks remains inadequately characterized.

## 5. Mitochondrial Dynamics and Their Regulatory Mechanisms in Oocyte Maturation

**Figure 4:**
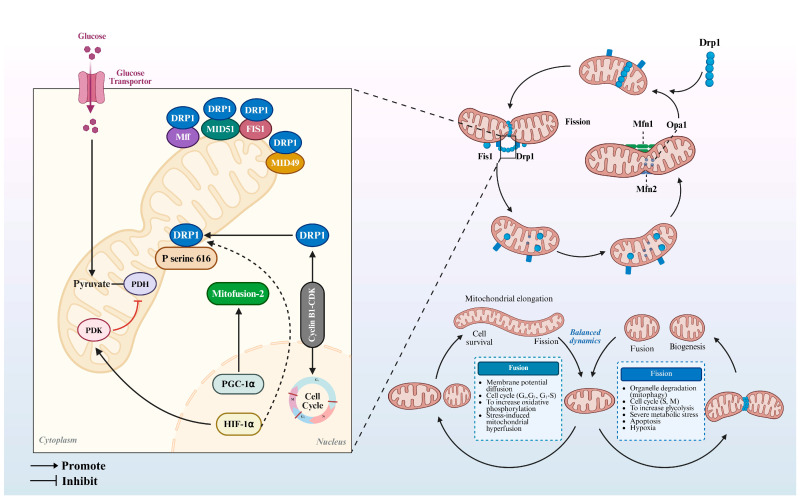
Mitochondrial dynamics: core regulatory machinery and modulatory determinants. Created in BioRender. https://www.biorender.com/ (accessed on 10 April 2025).

As highly dynamic organelles, mitochondria undergo continuous fusion–fission cycles to regulate their morphology, size, and subcellular distribution. This dynamic process thereby ensures precise ATP flux regulation for cellular processes critical to oocyte maturation [85,86]. Key regulators include mitofusin 1/2 (MFN1/2) and optic atrophy 1 (OPA1) for mitochondrial fusion, while mitochondrial dynamics-related protein 1 (DRP1) governs fission (Figure 4) [87]. During oocyte transition to metaphase I (MI stage), mitochondria stage-specifically assemble into larger clusters with perinuclear polarization, maintaining spatial segregation from the spindle apparatus in a spindle apparatus-independent manner. Notably, individual and small mitochondrial clusters persist in even distribution despite reduced abundance compared to the premeiotic stage. By metaphase II (MII), mitochondrial clustering intensifies, forming larger aggregates than those in MI oocytes. Recent studies demonstrate that overexpression of mitochondrial fusion proteins induces cytoplasmic mitochondrial aggregation, disrupting spindle organization and spatiotemporal endoplasmic reticulum distribution [85]. Furthermore, oocyte-specific DRP1 knockdown impairs calcium signaling and cell–cell communication, resulting in follicular developmental defects and ovulation abnormalities [88].

Emerging evidence implicates mitochondria-associated membranes (MAMs) and mitochondria-ER contacts (MERCs) in cytoplasmic maturation deficits of oocytes from obese mice, potentially mediated by elevated Ca^2+^ levels, with significant differences (*p* < 0.05) compared to the control group [89,90]. Paradoxically, elevated perichromosomal mitochondrial Ca^2+^ may enhance mitochondrial energy output to support calmodulin-responsive spindle formation [91,92]. During reproductive aging in C. elegans, increased GTP-dependent mitochondrial fission near the inner membrane may drive EAT-3-mediated mitochondrial fusion, with this dynamic imbalance contributing to oocyte quality decline [93]. Sirt3 deficiency exacerbates superoxide accumulation and spindle disassembly in aged oocytes, accompanied by mitochondrial dysfunction characterized by uneven distribution, reduced membrane potential, and decreased mtDNA content. Studies reveal that compared with that of the wild type, mitochondrial membrane potential was significantly reduced in the aged group (*p* < 0.01), and mtDNA copy number was significantly reduced (*p* < 0.01) [94]. Emerging evidence highlights the critical role of optic atrophy 1 (OPA1). Defects in this inner mitochondrial membrane protein promote excessive mitochondrial fission and dysfunction [95,96]. Similarly, deoxyguanosine kinase (DGUOK) deficiency in murine oocytes leads to aberrant mitochondrial dynamics and marked reduction in mitochondrial DNA (mtDNA) copy number (*p* < 0.05) [97,98]. A study by further reports that deletion of mitochondrial outer membrane proteins MIGA1/2 induces cytoplasmic mitochondrial dynamics defects, potentially due to significantly elevated (*p* < 0.05) intra-mitochondrial ROS levels, reduced Δ*Ψm*, and diminished mtDNA content compared to the wild type [99,100]. Additionally, hyperthermia impairs bovine oocyte embryonic development, with recent studies confirming its adverse effects on mitochondrial dynamics in porcine oocytes [101]. In mouse ovaries, Sirt3 deficiency diminishes mitochondrial respiratory chain complex activity and downregulates key proteins associated with mitochondrial fusion (OPA1, *p* < 0.001; MFN2, *p* < 0.05) and fission (DRP1, *p* < 0.001; FIS1, *p* < 0.001), ultimately compromising overall mitochondrial functionality [94]. These findings emphasize the crucial role of SIRT3 in maintaining ovarian follicle reserves and oocyte quality with aging [94,102]. Mfn2 deletion induces profound transcriptomic alterations in mouse oocytes, disrupting mitochondrial and ER homeostasis, while Mfn2-deficient oocytes exhibit mitochondrial functional impairment [103,104]. Furthermore, the knockdown of activating transcription factor 5 (ATF5) in mouse oocytes disrupts mitochondrial dynamics. ATF5 is involved in regulating vitrified oocytes through the cAMP-PKA and PI3K/AKT pathways [105,106].

In summary, mitochondria preserve their functional integrity through ongoing cycles of fission and fusion, which are closely linked to lipid metabolism and ATP production. Although current research primarily concentrates on mitochondrial dynamics regulators and their connection to oocyte maturation, the molecular interplay between mitochondrial dynamics and oocyte maturation has been insufficiently investigated.

## 6. Molecular Mechanisms by Which Mitophagy Regulates Oocyte Maturation

**Figure 5:**
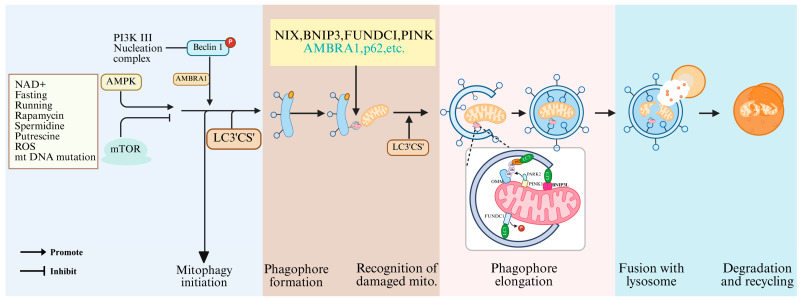
Mitophagy-regulated signaling pathways. Created in BioRender. https://www.biorender.com/ (accessed on 10 April 2025).

Autophagy is triggered by diverse cellular stress conditions, including nutrient deprivation, oxidative stress, pathogenic infection, or inflammatory stimuli, as extensively documented [107,108]. This vital process unfolds through three distinct yet interconnected mechanisms: chaperone-mediated autophagy, microautophagy, and macroautophagy. Mitophagy, a specialized form of macroautophagy, selectively degrades mitochondria through sequential steps involving depolarization and loss of Δ*Ψm* in damaged mitochondria, encapsulation of mitochondria by autophagosomes to form mitophagosomes, fusion of mitophagosomes with lysosomes, and lysosomal degradation of mitochondrial components [109,110]. Mitophagy mechanisms are broadly classified into ubiquitin-dependent and ubiquitin-independent pathways. The ubiquitin-dependent pathway, exemplified by the well-characterized PINK1/Parkin cascade, involves extensive ubiquitination of mitochondrial surface proteins to trigger degradation [58,111]. In this process, Δ*Ψm* collapse in damaged mitochondria prevents PINK1 translocation to the inner mitochondrial membrane, leading to its stabilization on the outer membrane surface [112]. PINK1 undergoes autophosphorylation and recruits/activates Parkin, an E3 ubiquitin ligase that ubiquitinates mitochondrial substrates. Subsequent binding of ubiquitinated proteins to microtubule-associated protein 1 light chain 3 (LC3)adaptors initiates autophagosome formation (Figure 5) [112], ultimately eliminating ROS-producing mitochondria to prevent oxidative DNA damage, spindle defects, and apoptosis, thereby ensuring oocyte survival during meiotic progression (GVBD to MII) and cytoplasmic maturation.

Mitophagy regulation shares similarities with mitochondrial biogenesis and dynamics. Resveratrol [113], CNP [114], SIRT1/2 [115,116], OPA1 [95,96], and rotenone collectively influence mitophagy. Resveratrol and OPA1 activate the PINK1/Parkin pathway, elevating mitophagy levels. Specifically, resveratrol upregulates PINK1, Parkin, and LC3B-II while downregulating MFN2, VDAC1, and p62 [117]. CNP ameliorates oxidative damage and suppresses excessive PINK1/Parkin-mediated mitophagy, restoring mitochondrial oxidative phosphorylation and rescuing aging-related defects in murine oocytes [114]. Melatonin co-treatment mitigates gossypol-induced damage in porcine oocytes by upregulating PINK1, Parkin, and LC3 expression while downregulating p62, a protective mechanism linked to SIRT1 activation and enhanced mitophagy [115]. Conversely, SIRT2 knockdown in ovine granulosa cells reduces oocyte maturation rates and elevates mitophagy [107,118]. Rotenone treatment in porcine oocytes significantly increases LC3 and active-caspase 3 levels, indicating that rotenone-induced mitochondrial dysfunction triggers compensatory mitophagy but ultimately promotes apoptosis [73]. Studies in porcine postovulatory aging (POA) models demonstrate that DRP1-mediated mitochondrial fission and mitophagy activation are essential for oocyte quality maintenance. β-Nicotinamide mononucleotide (NMN)-mediated activation of nicotinamide adenine dinucleotide (NAD) not only enhances oocyte quality but also promotes mitophagy in POA models, establishing NAD biosynthesis as a key regulator of DRP1-dependent mitochondrial morphology and homeostasis [119]. In Drosophila oocytes, fasting downregulates the DIP1-Clueless pathway, elevating stable intron-derived RNA sisR-1 expression. Mechanistically, sisR-1 localizes to mitochondrial aggregates, inhibits polyubiquitination of outer membrane protein Porin/VDAC1, and suppresses p62-mediated mitophagy [120,121]. Refeeding reverses fasting-induced sisR-1 elevation, restoring mitophagy, oogenesis, and oocyte quality. Activation of the PRKN-mediated mitophagy pathway coincides with meiotic arrest at metaphase I, linked to reduced RAB7 activity. Notably, microinjection of constitutively active RAB7Q67L mRNA or treatment with RAB7 activator ML098 rescues mitophagy–chromosome stability defects in CCCP-treated oocytes, highlighting RAB7 activators as potential interventions for age-related oocyte quality decline [122,123]. In rat ovaries, dehydroepiandrosterone (DHEA) attenuates significantly upregulated expression of mitophagy-related markers (Pink1, Parkin, BNIP3L, LC3, AMPK, SIRT1) and AMPK phosphorylation in diminished ovarian reserve (DOR) models, effectively suppressing AMPK-SIRT1-driven mitophagy and improving ovarian reserve [124,125]. Experimental studies demonstrate that spermidine supplementation improves oocyte quality and reproductive performance in aged female mice by enhancing mitochondrial autophagy pathways through upregulated LC3 expression [126].

The BNIP3/NIX pathway has emerged as a prominent research focus in mitochondrial quality control. As an outer mitochondrial membrane protein within the BH3-only subfamily of the BCL2 family, BNIP3L/NIX exerts dual regulatory functions in autophagy processes [127,128]. Mechanistically, BNIP3L initiates autophagosome formation by dissociating the BCL2-BECN1 complex and liberating BECN1. This autophagy-inducing activity can be pharmacologically replicated using BH3 domain mimetic small molecules. Furthermore, BNIP3L demonstrates MTORC1 inhibitory capacity to activate autophagy pathways [128]. Structural analysis reveals that BNIP3 contains evolutionarily conserved LC3-interacting region (LIR) motifs at its N-terminus, enabling direct LC3 recognition and binding to trigger mitophagy. Post-translational modifications critically regulate BNIP3L-mediated mitochondrial clearance: phosphorylation events predominantly mediate mitophagy activation, whereas ubiquitination provides an alternative pathway for selective removal of BNIP3L-tagged mitochondria [128,129].

FUNDC1 serves as a crucial mitochondrial receptor protein that mediates Parkin-independent mitophagy in mammalian cells during hypoxic stress. This process kicks off with a dynamic interplay between FUNDC1’s LC3-interacting region (LIR) domain and LC3 proteins anchored on autophagosomal membranes, orchestrating the precision-guided engulfment of damaged mitochondria [130]. Furthermore, the mitophagic activity of FUNDC1 is precisely modulated by dynamic phosphorylation events at specific serine residues (Ser13 and Ser17), where different kinase pathways exert opposing regulatory effects—phosphorylation by Unc-51-like kinase 1 (ULK1) enhances mitophagy initiation while phosphorylation by casein kinase 2 (CK2) suppresses this process [131,132,133].

Although the PINK1/Parkin pathway has been extensively characterized in mitophagy regulation, the intricate molecular crosstalk between mitophagy, mitochondrial quality control networks, and oocyte maturation remains shrouded in ambiguity, demanding rigorous mechanistic investigations to unravel these complex interdependencies.

## 7. Epigenetic Modifications in Mitochondrial Quality Control: Molecular Mechanisms and Functional Consequences

Most mitochondrial localization proteins are related to the transcription of nuclear-encoded genes. After the DNA in the nucleus is expressed as a protein, it is transported to the mitochondria to participate in MQC. In terms of genome methylation, studies have found that in the liver, DNA Methyltransferase 1 (DNMT1) is closely related to mitochondrial biogenesis. DNMT3B mediates the hypermethylation of the mitochondrial biogenesis regulatory factor PGC-1α gene [134]. In addition, as was found in human mesenchymal stem cells, DNMT3A is associated with methylation of the PGC-1α promoter, and methylation of its promoter region inhibits PGC-1α expression. Reduced PGC-1α expression leads to increased expression of the mitochondrial fission marker Drp1 [134], which in turn promotes the mitochondrial fission process. Increased mitochondrial fission is associated with decreased mitochondrial function [135]. In the heart, oxidative stress reduces DNMT1 activity, leading to hypomethylation of mitochondrial genes related to mitochondrial biogenesis and mitochondrial dynamics, such as PGC-1α, NRF-1, and TFAM. This imbalance ultimately leads to mitochondrial damage [136,137]. Significantly reduced methylation levels were observed in the mitochondrial DNA promoters HSP1 and HSP2 within the rat hippocampus, resulting in impaired TFAM binding and a consequent diminished capacity for mitochondrial biogenesis [138,139]. In the placenta, methylation of the MT-RNR1 region of mtDNA has been implicated in driving heightened mitophagy [140,141]. Within dopaminergic neurons, hypermethylation of the Ambra1 gene may disrupt Parkin binding, amplify LC3B expression, and ultimately exacerbate mitochondrial dysfunction and neuronal damage both in vitro and in vivo through the Am-bra1/Parkin/LC3B mitophagy cascade [142,143]. Studies in rat hearts demonstrated that DNMT1 enhances ETS1 expression through methylation of the miR-152-3p promoter region, stimulates RhoH transcriptional activation, and suppresses mitochondrial autophagy in H9c2 cells [144,145]. In addition, DNMT3A-mediated hypermethylation of the secreted frizzled-related protein 3 promoter is associated with excessive mitochondrial fission [146,147].

In the realm of RNA methylation, the RNA demethylase human AlkB homolog 5 (ALKBH5) actively diminishes significantly mitochondrial membrane potential and oxygen consumption rates, while suppressing mitochondrial fission and proliferation processes [148]. Mechanistically, ALKBH5 catalyzes the removal of N6-methyladenosine (m6A) methylation marks within the 3′ untranslated region of Drp1 transcripts, thereby downregulating Drp1 protein levels significantly and attenuating mitochondrial fission processes [134]. Furthermore, studies demonstrate that overexpression of the human mitochondrial transcription factor B1 induces pronounced hypermethylation of 12S rRNA, culminating in significant mitochondrial biogenesis abnormalities [149,150]. Research has revealed that m6A RNA methylation plays a pivotal role in regulating mitochondrial function by facilitating the translation of nuclear-encoded mitochondrial complex subunit RNAs [151]. Mettl14 knockout-induced m6A deficiency significantly reduces metabolites related to energy metabolism [152]. Tu et al. (2023) had revealed that METTL3 downregulation suppresses mitochondrial fission [153]. This process is driven by METTL3-mediated m6A methylation of the long non-coding RNA GAS5, which accelerates its degradation [153]. Intriguingly, GAS5 exhibits direct molecular interaction with Drp1, a pivotal mitochondrial fission protein, and its overexpression potently blocks Drp1-dependent mitochondrial fragmentation [154]. Furthermore, studies have demonstrated that m6A methylation at the A2212 site within OPA1’s coding sequence enhances mRNA stability, thereby promoting mitochondrial fusion [155]. Remarkably, therapeutic targeting of m6A modification through either the methyl-transferase inhibitor STM2457 or the dm6A CRISPR system produces profound inhibitory effects on mitochondrial fusion processes [156].

In the regulatory landscape of histone modification, protein arginine methyltransferase 5 (PRMT5) emerges as a crucial epigenetic modulator that catalyzes the symmetric di-methylation of arginine residues at histone H4R3, H3R8, and H2AR3 loci [134]. This enzymatic activity mechanistically establishes transcriptional repression, effectively downregulating PGC-1α expression through chromatin remodeling pathways. PRMT5 silencing or complete deletion significantly boosts PGC-1α expression and stimulates mitochondrial biogenesis [134]. Studies have demonstrated that endurance exercise training in the rat gastrocnemius muscle modulates histone methylation patterns (H3K4me3 and/or H3K27me3) within the promoter regions of the PGC-1α gene via specific epigenetic regulatory mechanisms, leading to a significant upregulation of both PGC-1α mRNA and protein expression levels (*p* < 0.01), which consequently enhances mitochondrial biogenesis [157,158]. Mitochondrial biogenesis is enhanced by histone H3 acetylation, in particular H3 lysine 14 acetylation (H3K14ac) [159,160]. In mice, succinate supplementation was found to increase permissive histone succinylation and H3K4me3 modification in the PGC-1α promoter, which correlated with higher PGC-1α expression [161]. In addition, SET And MYND Domain Containing 3 (SMYD3) was found to activate methylene tetrahydrofolate dehydrogenase 1-like by catalyzing the trimethylation of lysine at position 4 of histone subunit 3 (H3K4me3) [162], leading to the accumulation of the intracellular metabolites, which increases the expression of autophagy-related proteins LC3, PINK1, and Parkin and promotes mitochondrial autophagy [163].

Furthermore, mitochondrial–nuclear crosstalk profoundly regulates nuclear epigenetic modifications, mitochondrial functionality, and downstream metabolic byproducts, collectively governing oocyte maturation. Acetyl-CoA, a pivotal node in the tricarboxylic acid (TCA) cycle, is generated from mitochondrial precursors, including pyruvate, amino acids, and fatty acids. It mediates nuclear histone acetylation catalyzed by histone acetyltransferases (HATs), which neutralizes lysine residue charges to relax chromatin structure, facilitating transcription factor binding and gene regulation [164]. Studies reveal that the α-ketoglutarate dehydrogenase complex interacts with Jumonji C (JmjC)-domain-containing histone demethylases, competitively depleting α-ketoglutarate to inhibit JMJ activity, thereby modulating DNA/histone methylation levels, α-ketoglutarate concentration, and TCA cycle flux [165]. NAD^+^ and flavin adenine dinucleotide (FAD), pivotal coenzymes bridging the TCA cycle to electron transport via oxidative phosphorylation, play central roles in epigenetic regulation: FAD acts as an indispensable cofactor for lysine-specific demethylases, mitochondrial activity tightly regulating its bioavailability and redox balance, while NAD^+^ fuels the deacetylase activity of Sirtuins (SIRTs), which orchestrate histone acetylation dynamics [166]. Succinyl-CoA, synthesized from α-ketoglutarate and CoA via the α-ketoglutarate dehydrogenase complex (OGDH), is converted to succinate by succinyl-CoA synthetase. Succinate exits mitochondria through the dicarboxylate carrier SLC25A10 to regulate histone lysine succinylation [167]. Mitochondrial catabolism of tryptophan and lysine converges at α-ketoglutarate, which OGDH converts to succinyl-CoA. The FAD-dependent succinyl-CoA dehydrogenase subsequently catalyzes the conversion into an enzyme-bound intermediate, simultaneously yielding FADH_2_ to fuel electron transport [168]. Glutaryl-CoA is then decarboxylated into crotonyl-CoA, which cascades through successive metabolic transformations—becoming β-hydroxybutyryl-CoA, then acetoacetyl-CoA, before culminating in acetyl-CoA that integrates seamlessly into the TCA cycle [168]. Despite its low abundance, crotonyl-CoA-mediated histone lysine crotonylation functions as a positive transcriptional regulator and marks chromatin active regions [169,170].

**Figure 6:**
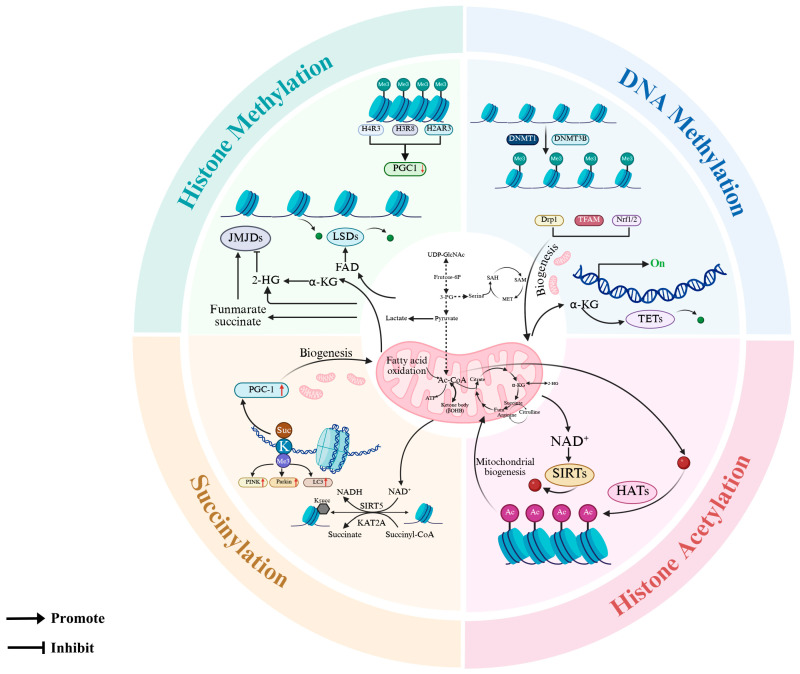
Mitochondria coordinate epigenetic modifications and quality control through metabolite-mediated regulation. Mitochondrial metabolites, such as α-KG, acetyl-CoA, NAD^+^, and succinate, serve as key substrates or cofactors that directly modify chromatin and histones. Created in BioRender. https://www.biorender.com/ (accessed on 10 April 2025).

In summary, epigenetic mechanisms emerge as a central regulatory nexus governing mitochondrial biogenesis, orchestrating dynamic structural adaptations, and finetuning quality control through mitophagy. The majority of these effects arise following epigenetic modifications in nuclear-encoded genes, which ultimately impact MQC proteins and consequently destabilize the MQC network. Intriguingly, certain epigenetic alterations directly target mtDNA or induce structural changes in MQC proteins (Figure 6). Nevertheless, the precise molecular mechanisms governing how epigenetic modifications steer the MQC network’s functionality within the realm of reproductive biology—particularly in shaping oocyte development and quality—remain profoundly enigmatic.

## 8. Methodological Advances in Mitochondrial Quality Control Research of Oocytes

Given that oocytes are significantly fewer in number compared to somatic cells, detecting and tracking molecular changes within MQCs poses a formidable challenge when relying on conventional methodologies. To propel this field forward, it is imperative to integrate cutting-edge multimodal approaches—such as dynamic subcellular organelle tracking via live-cell imaging, coupled with spatially resolved single-cell multi-omics (transcriptomics, metabolomics, proteomics, and epigenomics)—to holistically address persistent technological constraints. When it comes to high-resolution live-cell imaging for observing subcellular organelles, conventional visible light microscopy remains constrained by the diffraction limit, achieving maximum resolutions between 200 and 250 nanometers [171]. Taking mitochondria as an example, the width of the crest is often 20–100 nm. Therefore, super-resolution microscopy techniques have emerged, including wide-field imaging methods based on spatial spectrum expansion (the representative technology is structured illumination microscopy (SIM)), the laser scanning imaging method based on point spread function (PSF) compression (the representative technology is stimulated emission depletion (STED)), single-molecule localization microscopy (SMLM) based on single-molecule localization imaging method (the representative technology is stochastic optical reconstruction microscopy (STORM)), and photoactivated localization microscopy (PALM) [172]. SIM technology does not require special fluorescent labeling, requires low illumination light intensity, has low photobleaching and photodamage to the sample, and is fast, making it suitable for live-cell imaging, especially long-term live-cell imaging. STED technology relies on the principle of using two lasers, one to excite the fluorescent molecules and the other, the STED laser, to suppress the emission of fluorescent groups located outside the excitation center [173]. As a result, a smaller point spread function is obtained, and its optical resolution is improved. The principle of SMLM technology is to collect sparse fluorescent molecules multiple times. The result of synthetic single-molecule localization is the distribution of fluorescent molecules in the entire field of view, and the position information of these single molecules is superimposed to achieve super-resolution imaging [174]. Combining the advantages of the three techniques, SIM technology is suitable for observing the interaction of mitochondria and other organelles and the division and fusion of mitochondria in a large field of view or with ultra-fast speed; STED technology can specifically label mitochondrial proteins and is suitable for dynamically observing changes in the ultrastructure of mitochondria in a small field of view; SMLM technology is suitable for observing protein localization and interactions. With the vigorous development of artificial intelligence and deep learning, in their latest research, et al. demonstrated a ratiometric HCIO probe AHOH that can simultaneously perform two-color imaging of lysosomes and mitochondria for monitoring mitochondrial autophagy [175]. In addition, a new near-infrared fluorescence probe, HBimmCue, has been developed. Combined with fluorescence lifetime imaging microscopy (FLIM), STED-FLIM imaging is used to reveal changes in mitochondrial membrane order, which in turn reflects mitochondrial function and cellular respiration levels [176,177].

In the realm of single-cell transcriptome sequencing, the single-cell transcriptome represents the comprehensive expression profile of all mRNAs within an individual cell at a specific timepoint, capturing the cell’s functional essence and biological state. Prominent technology packages, such as STRT-seq [178,179], Smart-seq [180,181], CEL-seq [182,183], Quartz-seq [184], and MATQ-seq [185,186], enable precise analysis, while leading platforms like 10x Genomics, BD Rhapsody, and Fluidigm C1 empower researchers with scalable, high-resolution solutions. Single-cell metabolomics is the qualitative and quantitative analysis of small molecule metabolites in biological samples. As direct products of the functional activities of organisms, changes in metabolites can reflect changes in phenotype. It is mainly divided into microscopy-based platforms, spectroscopy-based platforms, and mass spectrometry-based platforms. In comparison, mass spectrometry stands out among these techniques due to its high detection sensitivity, selectivity, wide detection range, fast analysis speed, and strong molecular structure identification capabilities. The process mainly includes single-cell sampling, content measurement, and data analysis. The workflow of single-cell metabolomics data analysis includes data preprocessing, metabolite annotation, statistical analysis, network and pathway analysis, and data visualization [187,188]. However, the single-cell metabolome is highly susceptible to variability in instrument operation and personnel expertise, which can significantly compromise the reliability of results. In terms of single-cell proteomics technology, the total protein content of a single cell is only 100–200 pg, and the amount in an oocyte is even less. Therefore, any loss will have an immeasurable impact, and it also puts forward more stringent requirements for the sensitivity and accuracy of the detection method. Single-cell proteomics can be divided into antibody-based detection methods such as mass spectrometry flow cytometry, PCR-based detection methods such as CITE-seq and REAP-seq, and mass spectrometry-based detection methods such as nanoPOTS. Recently, Yang et al. unveiled groundbreaking evidence identifying NADase CD38 as a central determinant governing age-related deterioration in oocyte quality [171]. At present, mass spectrometry has become the mainstream analytical tool for large-volume proteomics due to its high precision and high-throughput advantages [187]. In this study, researchers employed cutting-edge mass spectrometry-based single-cell proteomics technology, successfully identifying and quantifying a remarkable average of 1200 distinct proteins within each meticulously analyzed mouse oocyte sample [189,190]. In the realm of single-cell epigenomes, methylation predominantly arises at the fifth carbon position of cytosine derivatives, where critical molecular signatures like 5-hydroxymethylcytosine, 5-formylcytosine, 5-carboxycytosine, and m6A are forged through this biochemical artistry. Epigenome sequencing focuses on mining epigenetic information such as genome methylation and epigenetic information such as genome methylation and histone modification regulation. Common techniques encompass revolutionary single-nuclei ATAC-seq and precision-driven CUT&Tag [191]. Researchers constructed genome-wide maps of accessible chromatin in bovine oocytes and early embryos using ATAC-seq, revealing dynamic chromatin accessibility patterns during early embryonic development in cattle. The study demonstrated low chromatin accessibility in bovine oocytes and 2-/4-cell embryos, which markedly increased during the embryonic genome activation (EGA) phase and peaked in day 14 elongated embryos. Robust ATAC-seq signals were detected at both transcription start sites (TSSs) and transcription termination sites (TTSs) [192]. A separate mouse model study revealed that selective chromatin accessibility present in sperm and MII oocytes was extensively erased during early pronuclear stages through a protamine phosphorylation-dependent mechanism [193]. Studies in mouse models employing single-oocyte RNA sequencing with CUT&Tag analysis have demonstrated that DIS3 enzyme mediates transcriptional silencing through intergenic RNA degradation, a critical mechanism regulating chromatin condensation and meiotic resumption [194]. Separate investigations focusing on bovine germ cells have systematically analyzed chromatin accessibility features of activation markers (H3K4me3, H3K27ac) and repression markers (H3K9me3, H3K27me3) across developmental stages, including oocytes; two-cell, four-cell, and eight-cell embryos; morulae; blastocysts; inner cell masses; and trophectoderm tissues [195]. For example, CUT&Tag was used to identify differentially enriched regions of histone H3K23su in 5-resistant HCT15 cells by integrating with ATAC-seq and RNA sequencing data [196]. In summary, the technology for high-resolution observation of subcellular organelles in living cells and single-cell omics sequencing is becoming increasingly mature, which facilitates a better exploration of the link between cellular physiological activities and various intracellular organelles. However, there is no relatively complete report on the link between mitochondrial function and activity, the MQC network and oocyte maturation in bovine oocytes, and the underlying molecular mechanisms remain unclear.

## 9. Conclusions and Future Perspectives

Mammalian oocyte maturation plays a pivotal role in determining reproductive efficiency and advancing the livestock industry, positioning the enhancement of oocyte quality as a paramount objective in both animal husbandry and life sciences. Cutting-edge research has recently uncovered nutritional compounds and groundbreaking mitochondrial activators—such as resveratrol [197], ubiquinol-10 (the bioactive form of coenzyme Q10), and dehydroepiandrosterone—that demonstrate remarkable efficacy in bolstering mitochondrial efficiency and elevating the developmental potential of oocytes (Table 1) [107]. Coenzyme Q10 supplementation and nicotinamide mononucleotide intake improve ovarian reserve and mitochondrial performance in murine oocytes. Melatonin optimizes mitochondrial calcium homeostasis and membrane potential in human oocytes while upregulating antioxidant gene expression to enhance cytoplasmic maturation in bovine models [197]. The hydroxylamine derivative BGP-15 restores mitochondrial function in oocytes from obese mice, whereas quercetin increases mitochondrial quantity and reduces structural anomalies. Mitoquinone mesylate (MitoQ) effectively lowers ROS levels during bovine oocyte in vitro maturation [38]. Emerging evidence illuminates rhodioloside’s pivotal role in sustaining mitochondrial integrity within aged mouse oocytes, astaxanthin’s remarkable capacity to diminish ROS while revitalizing mitochondrial dynamics in bovine reproductive systems, and cycloastragenol’s groundbreaking telomerase-enhancing prowess that elevates bovine oocyte maturation to unprecedented levels [198,199,200,201].

Despite these advances, the molecular interplay between MQC networks and oocyte maturation remains poorly defined. Current research predominantly employs in vitro maturation models and protein-targeted analyses, leaving critical gaps in understanding how MQC mechanisms—particularly mitochondrial biogenesis, dynamics, and mitophagy—orchestrate maturation processes. Future investigations should prioritize elucidating how MQC-related physiological activities modulate the MQC network, the mechanistic links between MQC networks and bovine oocyte maturation, and the molecular pathways through which mitochondrial biogenesis, dynamics, and mitophagy coordinate to regulate maturation. Resolving these questions will establish targeted therapeutic strategies to optimize oocyte maturation and mitochondrial functionality.

## Figures and Tables

**Table 1:** Substances that improve mitochondrial function and oocyte quality.

Substances	Function	References
Resveratrol	Meiotic spindle stabilization; intracellular ROS reduction; mitochondrial biogenesis activation; cytoplasmic maturation enhancement; blastocyst formation promotion	[107,197,202,203,204,205,206]
Coenzyme Q10	Suppresses ROS generation; mitigates oxidative stress-induced apoptosis; facilitates nuclear maturation; enhances oocyte quality; improves embryonic developmental competence; alleviates oxidative stress; reinforces mitochondrial function; accelerates developmental progression; promotes blastocyst formation rates	[207,208,209,210,211]
Nicotinamide Mononucleotide	ROS accumulation attenuation; meiotic chromosomal misalignment correction; mitochondrial membrane potential restoration; ATP synthesis augmentation; mitochondrial autophagy activation; oocyte maturation rate elevation; spindle assembly fidelity preservation; NAD^+^ pool replenishment in cumulus–oocyte complexes	[212,213]
Melatonin	ROS scavenging system activation (glutathione/antioxidant genes); mitochondrial architecture–function coordination; epigenetic regulation maintenance (methylation/hydroxymethylation)	[197,214,215,216,217]
α Lipoic Acid	Mitochondrial functional boost (activity/mtDNA); transcriptional fine-tuning; oxidative damage neutralization	[218,219]
L-carnitine	Metabolic flux optimization (fatty acid/glucose/respiratory chain); oxidative stress–apoptosis axis suppression; oocyte rejuvenation triad (glutathione/membrane potential/cytoplasmic maturation)	[220,221]

## Data Availability

Not applicable.
